# Astrocyte development—More questions than answers

**DOI:** 10.3389/fcell.2023.1063843

**Published:** 2023-03-27

**Authors:** Kathryn M. Markey, Jillian C. Saunders, Jana Smuts, Catherine R. von Reyn, A. Denise R. Garcia

**Affiliations:** ^1^ Department of Biology, Drexel University, Philadelphia, PA, United States; ^2^ Department of Neurobiology and Anatomy, Drexel University, Philadelphia, PA, United States; ^3^ School of Biomedical Engineering, Science and Health Systems, Drexel University, Philadelphia, PA, United States

**Keywords:** astrocyte, glia, development, *Drosophila*, astrocyte-like glia, neuropil glia, last modified: 10/7/22 12:36 p.m. p. 2

## Abstract

The past 15–20 years has seen a remarkable shift in our understanding of astrocyte contributions to central nervous system (CNS) function. Astrocytes have emerged from the shadows of neuroscience and are now recognized as key elements in a broad array of CNS functions. Astrocytes comprise a substantial fraction of cells in the human CNS. Nevertheless, fundamental questions surrounding their basic biology remain poorly understood. While recent studies have revealed a diversity of essential roles in CNS function, from synapse formation and function to blood brain barrier maintenance, fundamental mechanisms of astrocyte development, including their expansion, migration, and maturation, remain to be elucidated. The coincident development of astrocytes and synapses highlights the need to better understand astrocyte development and will facilitate novel strategies for addressing neurodevelopmental and neurological dysfunction. In this review, we provide an overview of the current understanding of astrocyte development, focusing primarily on mammalian astrocytes and highlight outstanding questions that remain to be addressed. We also include an overview of *Drosophila* glial development, emphasizing astrocyte-like glia given their close anatomical and functional association with synapses. *Drosophila* offer an array of sophisticated molecular genetic tools and they remain a powerful model for elucidating fundamental cellular and molecular mechanisms governing astrocyte development. Understanding the parallels and distinctions between astrocyte development in *Drosophila* and vertebrates will enable investigators to leverage the strengths of each model system to gain new insights into astrocyte function.

## Introduction

Astrocytes comprise a diverse population of cells responsible for a broad array of functions in the nervous system. Nevertheless, our understanding of fundamental principles of astrocyte biology, including their development, remains far behind that of other glial cell populations in the nervous system, including oligodendrocytes and Schwann cells (Jessen and Mirsky, 2005). Astrocyte progenitors are specified during embryonic development. However, their production, migration, and maturation occur predominantly during the first three to four weeks of postnatal development, coincident with synapse formation (Sauvageot and Stiles, 2002; Farhy-Tselnicker and Allen, 2018). Astrocytes are required for synapse formation and a growing number of studies further demonstrate the role of astrocytes in modulating synaptic function and behavior across several invertebrate and vertebrate species (for review, see Freeman, 2015; Nagai et al., 2021). Understanding the mechanisms governing the production, migration, and maturation of astrocytes will not only facilitate novel insight into the establishment of neural circuits underlying behavior but will further enable the development of novel therapeutic strategies for treating neurological dysfunction arising from neurodevelopmental disorders or injury.

**FIGURE 1:**
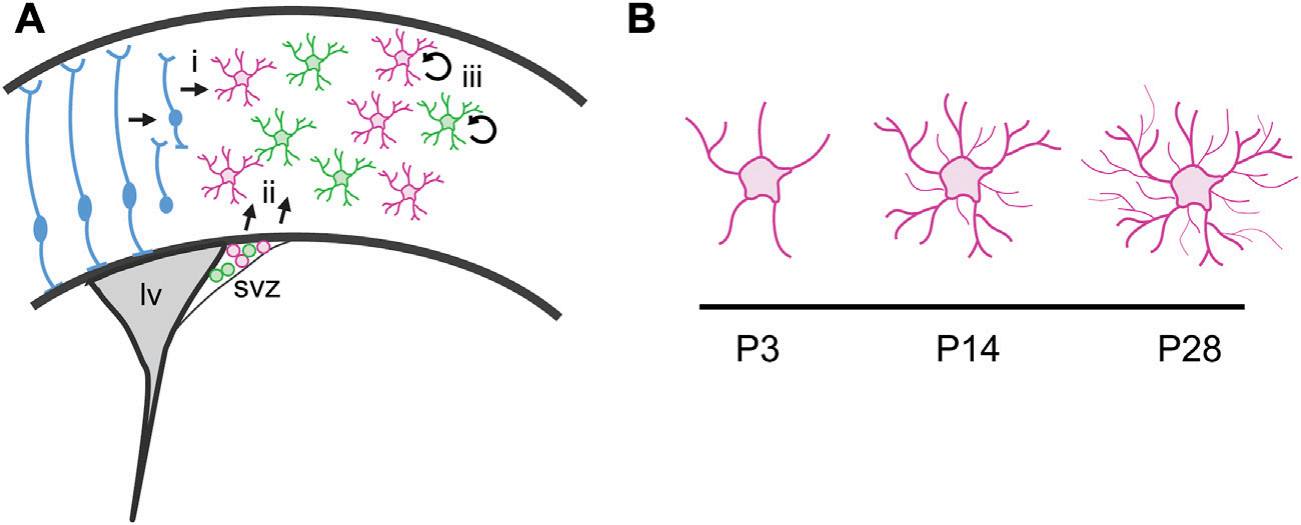
Astrocyte production and maturation during postnatal development. **(A)** Cortical astrocytes are derived from two progenitor cell sources. (i) Radial glia transform into cortical astrocytes and also serve as scaffolding for migration of immature astrocyte progenitor cells. (ii) A second pool of astrocyte progenitor cells reside in the dorsolateral corner of the subventricular zone (svz) at birth. The pool includes two molecularly distinct populations defined by Shh signaling. These cells migrate into the cortex during the first week of postnatal development and continue to proliferate locally, expanding the astrocyte population. (iii) Local proliferation of mature, differentiated astrocytes in the cortex expands the astrocyte population. lv, lateral ventricle. **(B)** Astrocytes undergo dramatic morphogenesis during the first few weeks after birth, elaborating a complex network of fine processes that interact with synapses.

Much of our understanding about astrocyte development is derived from classic neuroanatomical studies of the mammalian neocortex. At birth, radial glia begin retracting their processes and transform into protoplasmic astrocytes (Figure 1). Immature astrocytes then undergo morphological and functional maturation over the first few weeks of postnatal development. Mature astrocytes exhibit diverse morphologies, as well as differential expression of ion channels (Kelley et al., 2018), transporters (Lehre et al., 1995), transcription factors (Garcia et al., 2010), signaling molecules and cell surface markers (John Lin et al., 2017; Blanco-Suarez et al., 2018; Batiuk et al., 2020; Bayraktar et al., 2020), among other genes (Westergard and Rothstein, 2020), raising the question of functional and molecular heterogeneity within this large population of cells in the CNS. Indeed, a growing number of RNA sequencing studies from many labs have identified multiple populations of mature astrocytes and their progenitors with defined transcriptional signatures throughout the rodent CNS (Chai et al., 2017; Boisvert et al., 2018; Loo et al., 2019; Cheng et al., 2022; Endo et al., 2022), among many others)*.* How such diversity might arise remains poorly understood. While local, neuronally derived cues have been shown to regulate expression of certain genes during postnatal development (Farmer et al., 2016; Hill et al., 2019; Bayraktar et al., 2020), there is evidence that lineage may also confer specific transcriptional signatures (Ohayon et al., 2021). Whether and how this sets the stage for interpreting local signals is not known.

Several fundamental principles of astrocyte development remain to be explored and defined. Among these are the identity of intermediate astrocyte progenitor cells that rapidly expand the astrocyte population, similar to transient amplifying cells or oligodendrocyte precursor cells that expand the neuronal and oligodendrocyte populations, respectively. What is the molecular identity of these cells and what are the underlying mechanisms regulating their cellular behaviors? What are the cues that regulate astrocyte migration to their final positions and are these cues intrinsic or environmentally derived? How do intrinsic and extrinsic cues coordinate to confer astrocyte identity and how fixed or plastic is this identity? In this review, we provide a broad overview of key molecular and cellular mechanisms that have been identified underlying various developmental events during astrocytogenesis. Because much of the focus on astrocyte development has historically centered on molecular and genetic programs regulating acquisition of astrocyte cell fate, our understanding of these mechanisms is much more advanced than that of later stages of development. For a comprehensive review of molecular regulation of astrocyte specification in the vertebrate CNS, we refer the reader to several excellent reviews, including (Rowitch and Kriegstein, 2010; Kanski et al., 2014; Takouda et al., 2017). Here, we aim to highlight later developmental events, such as migration, morphogenesis, and diversity where many opportunities remain for closing existing fundamental gaps in knowledge.

While much of our understanding of astrocyte development is derived primarily from rodent studies, there is a growing recognition and appreciation for glial cells in the *Drosophila* CNS whose functional properties suggest their analogous relationship to vertebrate astrocytes. Although the precise evolutionary relationship of *Drosophila* glia to mammalian astrocytes remains to be clearly defined (Freeman and Rowitch, 2013; Losada-Perez, 2018), similarities across functional, morphological, and molecular axes suggest analogous, if not homologous, relationships between these cells (Freeman and Rowitch, 2013; Yang and Jackson, 2019). Most notably, astrocyte-like glia (ALG) and ensheathing glia (EG) share several key phenotypical and functional properties with mammalian astrocytes, including complex morphologies, regulation of synapse formation and function, and phagocytosis of debris following injury (Awasaki et al., 2008; Freeman, 2015). Given the striking similarities between these cells in the *Drosophila* CNS and mammalian astrocytes, a greater understanding of fundamental principles underlying their development and maturation may yield important insights into mammalian astrocyte development. Here, we provide an overview of key principles of *Drosophila* glial development, focusing primarily on ALG. Although early events, such as the stereotyped production and migration of cells during embryonic and larval development differ substantially from vertebrate development, later events, such as morphogenesis and synaptic contact, have direct parallels with astrocyte development in rodents. We focus our discussion on these later events, and aim to highlight open questions and exciting areas for future investigation. For a more comprehensive discussion of *Drosophila* glia and their development, we refer the reader to several excellent reviews (Altenhein, 2015; Freeman, 2015).

## Astrocyte specification: Embryonic beginnings

Much of the history of the study of astrocyte development has focused largely on the specification of astrocyte progenitors from multipotent neural precursor cells in the developing embryo. In vertebrates, radial glia undergo a gliogenic switch and shift from producing neurons to glia. This gliogenic switch is orchestrated by a combination of both intrinsic and extrinsic molecular cues. Neural precursor cells isolated from the cortex of embryonic day 10 (E10) mice and cultured in clonal conditions initially produce neurons. Glia are not observed in the cultures until 10 days later (Qian et al., 2000), demonstrating that individual cells in the embryonic CNS possess intrinsic molecular programs that drive developmentally-dependent competence to generate neurons first, then glia. However, extrinsic molecular cues also play an instructive role in cell fate acquisition. Cortical progenitor cells isolated from E15 mouse brains produce astrocytes when they are grown on cortical slices from postnatal day 15 (P15) brains. However, when the cells are grown on E18 slices, the cells instead produce neurons (Morrow et al., 2001). Thus, the gliogenic competence of neural progenitor cells is a coordinated effort between intrinsic and extrinsic programs that include mechanisms to repress neuron production while promoting glial production.

A major challenge in understanding astrocyte development is the paucity of molecular identifiers of astrocytes and their progenitors in both vertebrates and invertebrates. Historically, vertebrate studies have relied heavily on expression of glial acidic fibrillary protein (GFAP) to identify the acquisition of an astrocytic fate by neural precursors. Although GFAP expression is low in most astrocytes in the healthy adult brain, fate mapping studies show that nearly all astrocytes in the mouse cortex and throughout the forebrain are derived from a GFAP-expressing progenitor cell (Tatsumi et al., 2018; Hill et al., 2019), supporting its utility as a tool for identifying astrocyte progenitors. Several key molecular signaling programs have been identified that regulate the switch from neurogenesis to gliogenesis. Among these is Jak/STAT signaling, which is stimulated by activation of ciliary neurotrophic factor-leukemia inhibitory factor (CNTF/LIF) receptors in progenitor cells (Bonni et al., 1997). Activation of Jak/STAT signaling leads to repression of proneuronal genes neurogenin-1 and neurogenin-2 and promotes GFAP expression (Bonni et al., 1997; Sun et al., 2001). As neural development proceeds, newborn neurons express the cytokine, cardiotrophin-1 (CT-1), which signals back to neural precursors to generate astrocytes (Barnabé-Heider et al., 2005). In addition, BMP signaling promotes the acquisition of astrocyte characteristics, including GFAP expression (Gross et al., 1996; Bonaguidi et al., 2005). This is accomplished by the generation of a molecular complex composed of SMAD/p300 and STAT which promotes the transcription of astrocytic genes including GFAP and S100β (Nakashima et al., 1999). *In vivo* conditional knockout of STAT3 using nestin-cre in the spinal cord and the cortex show a decrease of GFAP expression and an increase in proliferation of stem cells (Hong and Song, 2014; Su et al., 2020). Another key signaling pathway in astrocyte specification is Notch signaling. In vertebrates, activation of Notch upregulates GFAP expression (Ge et al., 2012). Notch activation promotes demethylation of the GFAP promoter (Namihira et al., 2009).

Much of the molecular signaling regulating the gliogenic switch was identified through studies *in vitro*. Studies *in vivo* have identified key transcription factors that coordinate the repression of neurogenic genes while promoting the acquisition of astrocyte cell fate. The first such transcription factor identified was NFIA (Deneen et al., 2006). Its expression *in vivo* is initially detected at embryonic day 11.5 in the mouse spinal cord, and coincides with expression of the glutamate transporter, GLAST, expressed in astrocyte progenitors (Shibata et al., 1997). NFIA interacts with Notch signaling to inhibit neurogenesis and is necessary and sufficient for gliogenesis (Deneen et al., 2006). Expression of another transcription factor, Sox9, precedes NFIA induction, and its deletion in the CNS severely impairs the production of astrocytes in the mouse spinal cord (Stolt et al., 2003). Later studies identified Sox9 as a regulator of NFIA expression in the mouse and chick spinal cord (Kang et al., 2012). Despite the essential requirement of NFIA and Sox9 expression for astrocyte production, neither are exclusive for astrocyte progenitors, suggesting that additional regulatory mechanisms are necessary to specify astrocytic identity. In the mouse neocortex, the transcriptional repressor, Zbtb20, cooperates with Sox9 and NFIA to drive gliogenesis. Overexpression of Zbtb20 by electroporation increases astrocyte production at the expense of neuron production in the mouse cortex, suggesting that it plays a role in the suppression of neuronal genes (Nagao et al., 2016). The critical role of NFIA in astrogliogenesis was further confirmed in a recent study deploying transcriptomic and epigenomic approaches to identify specific transcriptional programs and epigenetic states during astrogliogenesis (Tiwari et al., 2018). NFIA, together with ATF3 and RUNX2, acts at regulatory elements of genes associated with astrogliogenesis. Electroporation of each of these transcription factors alone, or in combination, in the mouse cortex, was sufficient to enhance astrocyte production at the expense of neurons.

## How does the brain achieve the proper number and distribution of astrocytes?

Much of the emphasis on astrocyte development has focused largely on glial specification events during embryonic development. However, astrocyte production, migration, and maturation occur largely during postembryonic development, peaking early in the perinatal period (Sauvageot and Stiles, 2002). Whereas the mechanisms governing the gliogenic switch during embryonic development are well characterized, the cellular and molecular mechanisms governing developmental events occurring during postembryonic stages, such as expansion of the progenitor population, migration of immature cells to their final positions, and maturation into fully differentiated astrocytes, are considerably less well understood.

Classic neuroanatomical studies from Rakic and colleagues demonstrated the gradual disappearance of radial glia together with the concomitant appearance of differentiated astrocytes in the primate neocortex (Schmechel and Rakic, 1979; Levitt and Rakic, 1980; Cameron and Rakic, 2004), suggesting that cortical astrocytes are derived from the transformation of radial glia (Figure 1). In the rodent, this process begins around birth and is largely complete by about P7 (Kriegstein and Alvarez-Buylla, 2009).

In addition to radial glia, the early rodent postnatal cortex also harbors a pool of glial progenitor cells residing in the dorsolateral corner of the subventricular zone (dlSVZ; Figure 1). These glial progenitors contribute both astrocytes and oligodendrocytes to the overlying cortex and white matter (Levison and Goldman, 1993; Zerlin et al., 1995) and can be identified by expression of BLBP and Sox9. These dlSVZ progenitors are thought to be derived from radial glia (Kriegstein and Alvarez-Buylla, 2009). Whether astrocytes derived directly from radial glial transformation or from dlSVZ progenitors represent molecularly or functionally distinct populations is not known. Addressing these questions will require developing approaches to molecularly dissect these astrocyte progenitor populations. Finally, a third pool of progenitor cells responsible for expanding the cortical astrocyte population has been reported (Ge et al., 2012). These “pioneer” cells are derived from radial glia and dlSVZ progenitors that colonize the cortex and acquire mature, differentiated characteristics before proliferating locally (Figure 1).

Astrocytes comprise a substantial number of cells in the mature brain. How expansion of astrocyte progenitors is accomplished to achieve the appropriate number of mature astrocytes is not well understood. Expansion of neuronal and oligodendrocyte populations is achieved through intermediate progenitor populations which each exhibit defined molecular signatures at distinct developmental stages (Bergles and Richardson, 2015; Hevner, 2019). Oligodendrocyte precursors and their progeny, for example, express Sox10 and Olig2, premyelinating oligodendrocyte progenitor cells express PDGFRa and NG2, and mature myelinating oligodendrocytes express myelin basic protein (MBP), myelin associated glycoprotein (MAG) and carbonic anhydrase 1 (CA1). In contrast, efforts to identify a defined pool of intermediate progenitor cells for vertebrate astrocytes are hindered by the inability to distinguish these cells molecularly from their neural precursor or their daughter cells. Temporal profiling of developing astrocytes in the spinal cord across several mid-embryonic to early postnatal time points identified the stage-specific gene expression profiles (Molofsky et al., 2013; Chaboub et al., 2016). It would be interesting to examine whether these genes identify specific astrocyte progenitor populations or play a role in regulating progenitor cell behaviors. As discussed above, local proliferation of “pioneer” astrocytes in the cortex has been suggested as one source of intermediate progenitors. However, their mature gene expression and morphological characteristics preclude their prospective identification and fail to distinguish them from their post-mitotic daughter cells. In the mouse spinal cord, a population of Aldh1L1 positive intermediate progenitor cells undergo transit amplification and are differentiated from radial glia progenitor cells (Tien et al., 2012). However, mature astrocytes also express Aldh1L1, leaving open the question of how to define the intermediate astrocyte progenitor pool and distinguish them from earlier gliogenic precursors and later mature astrocytes.

The molecular and cellular mechanisms that govern the precise distribution and localization of astrocytes are poorly defined. However, emerging evidence suggests that unlike neurons and oligodendrocytes which can migrate long distances to their final position, astrocytes appear to have considerably more limited migration potential. Sparse labeling of embryonic cortical precursors demonstrates that cortical astrocytes are distributed within a radially oriented column, mimicking the columnar organization of cortical neurons (Magavi et al., 2012). Indeed, fate mapping from regionally defined progenitor zones in the forebrain and spinal cord shows that astrocyte migration is limited to the radial territory directly overlying their progenitor domain (Hochstim et al., 2008; Tsai et al., 2012). While radial migration of astrocyte progenitors has been well documented, advanced 3D serial microscopy imaging approaches of large volumes combined with multiclonal lineage tracing demonstrated that individual clones also undergo wide dispersion in the cortex that is not strictly radial (Clavreul et al., 2019). This distribution is thought to arise from stochastic “seeding” of the cortex by astrocyte progenitor cells, followed by local proliferation and expansion. Because earlier studies analyzed individual tissue sections, it is possible that such large scale dispersion could not be easily detected. Indeed, imaging individual sections labeled by the same multiclonal lineage approach also produces clones with a columnar distribution (Loulier et al., 2014). Taken together, these studies demonstrate that cortical astrocytes use different strategies to migrate to their final positions. Because the methods used in these studies to mark progenitor cells relied on expression and recombination in non-specific astrocyte progenitor cells, whether these different migration strategies can be adopted by cells within a given lineage or whether this reflects an intrinsic heterogeneity between specific astroctyte progenitor cells is not known. Such heterogeneity may arise from underlying genetic programs or from temporal cues orchestrated by molecular signaling programs that remain to be identified.

While many clones contained cells with shared morphologies, some clones were comprised of both protoplasmic and pial astrocytes, cells with distinct morphological characteristics. These mixed clones suggest that local cues may drive the adoption of mature phenotypes. Consistent with this is the observation that cortical astrocytes exhibit distinct molecular signatures based on their position in upper or deep layers (Bayraktar et al., 2020). However, in mutants with cortical neuron layer defects, these defined astrocyte layer molecular signatures are disrupted, suggesting that neurons provide molecular cues that instruct molecular or positional characteristics. As discussed below, Sonic hedgehog (Shh) signaling has been identified in the cerebellum as one such cue. Elucidating additional cues and their underlying mechanisms will be important for advancing our understanding of astrocyte development.

## How do astrocytes mature?

The production and migration of immature astrocytes occurs predominantly during the first week of postnatal development, followed by the acquisition of mature functional and morphological characteristics that develops over the second—fourth postnatal weeks (Stogsdill et al., 2017). By comparing the transcriptional signatures of immature and mature human and mouse astrocytes, Li et al., observed that astrocyte-astrocyte contact inhibits proliferation and promotes the expression of genes associated with mature cells (Li et al., 2019). Notably, astrocyte conditioned media failed to produce the same degree of transcriptional maturation, suggesting that the signals promoting astrocyte maturation are not secreted but instead require direct contact between cells. Further experiments showed that inhibiting EGFR signaling promotes astrocyte maturation. Consistent with this, heparin-binding epidermal growth factor-like growth factor (HBEGF), a ligand for EGFR, inhibits astrocyte maturation whereas BMP signaling downregulates EGFR and promotes astrocyte maturation (Scholze et al., 2014). RNA-seq identified genes associated with cilium assembly as highly enriched and upregulated following HBEGF removal or EGFR inhibition. Sonic hedgehog signaling is transduced in primary cilia (Goetz et al., 2009). Thus, these observations are consistent with the identification of Shh activity in astrocyte progenitor cells (Gingrich et al., 2022) and suggests a role for Shh signaling in astrocyte maturation.

Mature astrocytes exhibit complex morphologies that mediate their intimate relationships with synapses. Understanding how these morphologies develop has been limited by a paucity of available tools to study morphogenesis. GFAP expression is limited to larger processes, exposing only the primary cytoarchitecture while the small branchlets associated with synapses remain concealed. Further, the full complexity of their morphological characteristics is lost when dissociated, and astrocytes in culture instead take on flat morphologies that lack the fine branchlets and leaflets associated with synapses and blood vessels. This limits the utility of *in vitro* approaches for investigating the mechanisms driving their morphogenesis. However, *in vivo* studies are beginning to illuminate such mechanisms. The development of technologies including intracellular dye injections or fluorescent reporters targeting the membrane or filling the cell, which can be delivered by transgenes or viral vectors, now enable full visualization of astrocyte morphologies *in vivo*. Application of these approaches reveals that astrocytes begin to exhibit numerous processes and complex morphologies by the end of the first week of postnatal development, with further elaboration of fine processes that produce their spongiform morphology between P14 to P28 (Figure 1; Bushong et al., 2004; Morel et al., 2014; Holt et al., 2019). The temporal coincidence between astrocyte maturation and the major period of synaptogenesis in the rodent brain, during the second—fourth weeks of postnatal development, together with the observations that astrocytes respond dynamically to neuronal activity (Cornell-Bell et al., 1990a; Cornell-Bell et al., 1990b; Hirrlinger et al., 2004; Genoud et al., 2006), have long led to speculation that synaptogenesis instructively cooperates with astrocyte morphogenesis. Consistent with this, loss of glutamatergic signaling through mGluR5 reduces the domain size of individual astrocytes, leading to a reduction in the number of synapses ensheathed by an astrocyte process (Morel et al., 2014). However, synaptic activity alone is not sufficient for astrocyte morphogenesis. Complete blockade of synaptic activity with TTX treatment does not impair the extension of astrocyte processes (Stogsdill et al., 2017), pointing to contact-mediated mechanisms underlying astrocyte morphogenesis. Astrocytes interact with neurons through neuroligins which bind to neurexins found on presynaptic terminals. Perturbation of this interaction *in vitro* and *in vivo* reduces astrocyte complexity and volume of processes infiltrating the neuropil (Stogsdill et al., 2017).

Growth factor signaling also plays a role in astrocyte morphogenesis. The growth factor, brain derived neurotropic factor (BDNF) regulates astrocyte morphogenesis *in vitro* and *in vivo* (Holt et al., 2019). A truncated isoform of the BDNF receptor, TrkB.T1 lacking the tyrosine kinase domain but containing an additional exon, is found predominantly in astrocytes. Loss of TrkB.T1 expression decreases astrocyte volume and morphological complexity *in vitro* and *in vivo*.

In zebrafish, astrocytes fail to elaborate complex morphologies in the absence of *fgfr3* and *fgfr4* (Chen et al., 2020). As discussed below, FGFR signaling is required for morphogenesis of *Drosophila* ALG, suggesting that the requirement for FGF signaling in astrocyte morphogenesis is conserved in vertebrates. This is supported by *in vitro* studies from rodent cells where addition of FGFs or overexpression of *fgfr3* promotes enhanced branch number and complexity (Perraud et al., 1988; Kang et al., 2014). Indeed, several members of the *fgf* family and their receptors are expressed in astrocytes (Miyake et al., 1996; Oh et al., 2003). Identifying the precise members of the *fgf* family and their mechanism of action on astrocyte morphogenesis would advance our understanding of their development and may shed new light on their interactions with synapses.

A key feature of astrocyte morphology is the complete tiling of the neuropil in a non-overlapping manner, ensuring synaptic coverage. While the precise function of this cellular behavior is not well understood, it is observed in both invertebrate and vertebrate astrocytes (Bushong et al., 2004; Halassa et al., 2007; Stork et al., 2014; Chen et al., 2020; Pogodalla et al., 2022) and severe injury or disease of the nervous system perturbs the distinct territories established by individual astrocytes (Sofroniew, 2014). Astrocytes express a large complement of cell adhesion molecules (Zhang et al., 2014; Hillen et al., 2018) and growing evidence shows that these molecules mediate homophilic and heterophilic interactions between other astrocytes and neurons that regulate astrocyte morphogenesis. Co-culturing astrocytes with neurons enhances morphological complexity (Holt et al., 2019). This effect requires physical contact with neurons as the addition of neuron-conditioned media fails to induce this complexity (Stogsdill et al., 2017). The hepatocyte cell adhesion molecule, hepaCAM, was recently identified as a molecular mediator of astrocyte-astrocyte interactions that regulates their complexity and territory volume (Baldwin et al., 2021). Selective perturbation of hepaCAM expression from a sparse population of cortical astrocytes increases territory overlap between neighboring cells with differential hepaCAM expression.

## *Drosophila* glia

**FIGURE 2:**
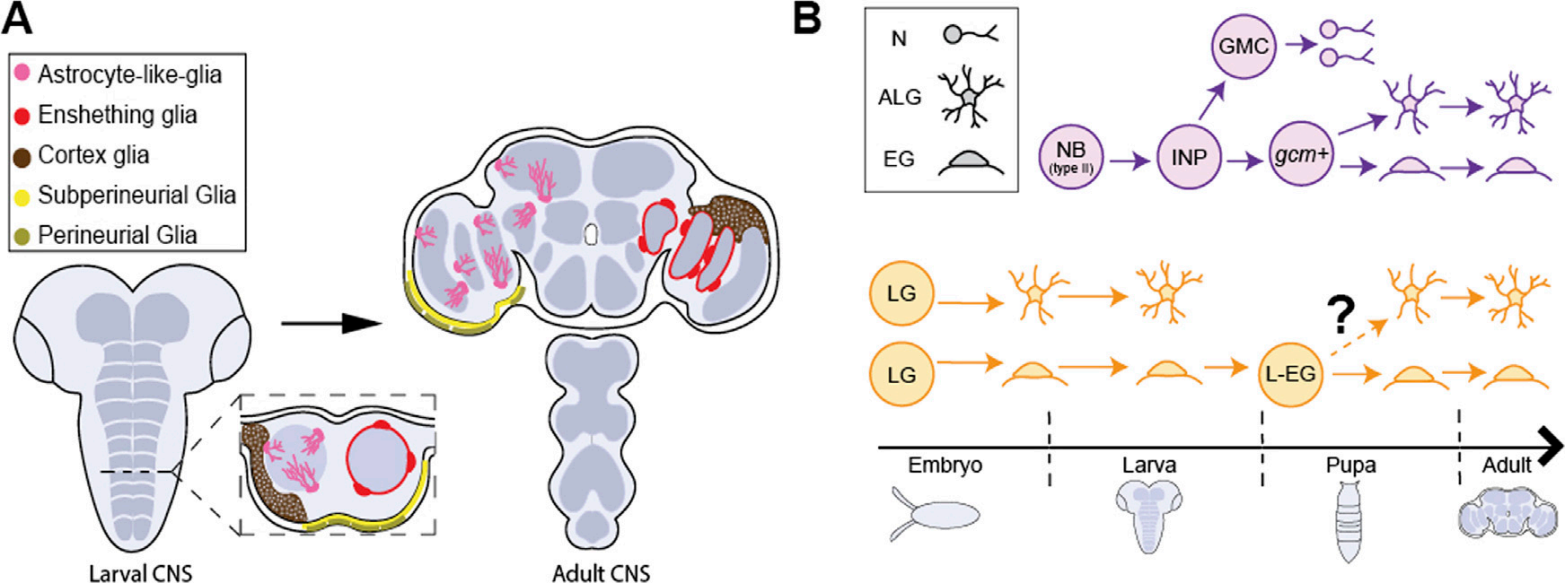
Glial cell types in *Drosophila* CNS. **(A)** The larval CNS (left) and adult CNS (right) with the 5 primary classes of glial cells, astrocyte-like glia (pink), ensheathing glia (red), cortex glia (brown), subperineural glia (yellow) and perineural glia (gold). **(B)** The adult CNS harbors neuropil glia (ALG and EG) arising from different progenitor populations at different stages in development. (Top, purple) Type II neuroblasts (Type II NB) generate bipotent intermediate neural progenitors (INPs) postembryonically. INPs generate ganglion mother cells (GMC) and *gcm*+ glial precursors which are responsible for generating a large fraction of ALG and EG after expansion. (Bottom, yellow) Embryonic glioblasts (not shown) generate longitudinal glia (LG) that are committed to generating ALG or EG cells in larva. It has been proposed that larval ALG are ultimately eliminated and surviving larval, EG (L-EG) dedifferentiate in pupa and become bipotent progenitors that can generate both ALG and EG for the adult CNS.

*Drosophila* offer powerful genetic and molecular tools, together with several connectome datasets (Zheng et al., 2018; Scheffer et al., 2020; Phelps et al., 2021) and well-characterized behaviors that can be exploited for advancing our understanding of astrocyte development and its role in neural circuits and behavior. *Drosophila* possess glial cells that, like mammalian astrocytes, play key roles in synapse formation and function, blood brain barrier function, phagocytosis of debris, and response to neural injury [Table 1; (Doherty et al., 2009; Limmer et al., 2014; Freeman, 2015)]. Interestingly, whereas these diverse functional roles are assigned to astrocytes in the mammalian CNS, glial cells in *Drosophila* performing these roles are assigned to several distinct glial cell classes. Surface glia, comprised of perineural and subperineural glia, cover the entire surface of the brain and perform blood-brain barrier functions (Figure 2; Awasaki et al., 2008; Stork et al., 2014). Cortex glia are found in the cell cortex of the CNS, ensheath neuronal soma, elicit transient calcium signaling, and provide phagocytic support during CNS development (Figure 2; Melom and Littleton, 2013; Coutinho-Budd et al., 2017; Nakano et al., 2019). Cortex glia form a dense meshwork, with each cell surrounding up to 100 individual neuronal soma (Kremer et al., 2017). Interestingly, mammalian astrocytes also surround neuronal soma, though at a considerably lower ratio of an average of 4 neurons per astrocyte (Halassa et al., 2007). Despite these intriguing morphological and functional properties, cortex glia are considerably understudied relative to other classes of *Drosophila* glia, and will not be discussed further here. Surrounding the neuropil are ensheathing glia (EG) and astrocyte-like glia (ALG) which together are known as neuropil glia (Figure 2; Pereanu et al., 2005). EG are found on the border of the neuropil where they define subcompartments in the CNS. Their processes interact with axon tracts and neuronal cell bodies and, EG participate in the phagocytosis of neuronal debris in the healthy and injured brain (Doherty et al., 2009). ALG are also found surrounding the neuropil. However, whereas, EG processes are largely excluded from the neuropil, ALG processes extend deep into the neuropil where they interact intimately with synapses and play key roles in their formation and modulating their function (Awasaki et al., 2008; Stork et al., 2014). *Drosophila* ALG maintain the balance of excitatory and inhibitory neurotransmitters and regulate synapse formation (Liu et al., 2014). ALG exhibit complex morphologies that tile the neuropil with little overlap in their processes, much like vertebrate astrocytes (Stork et al., 2014). Notably, some of the molecular signatures of ALG in larva and adult CNS are shared with vertebrate astrocytes, including the glutamate transporter, *Eaat1*, the GABA transporter, *gat*, and glutamine synthetase (Huang et al., 2015; Ng et al., 2016). As in the mammalian CNS, the development of ALG and synaptogenesis occur in parallel, highlighting their essential roles in synaptogenesis and synapse function (Muthukumar et al., 2014).

**TABLE 1:** Functional characteristics shared between vertebrate astrocytes and various classes of Drosophila glia. Neuropil glia, encompassing astrocyte-like and ensheathing glia, share many morphological and functional properties with vertebrate astrocytes.

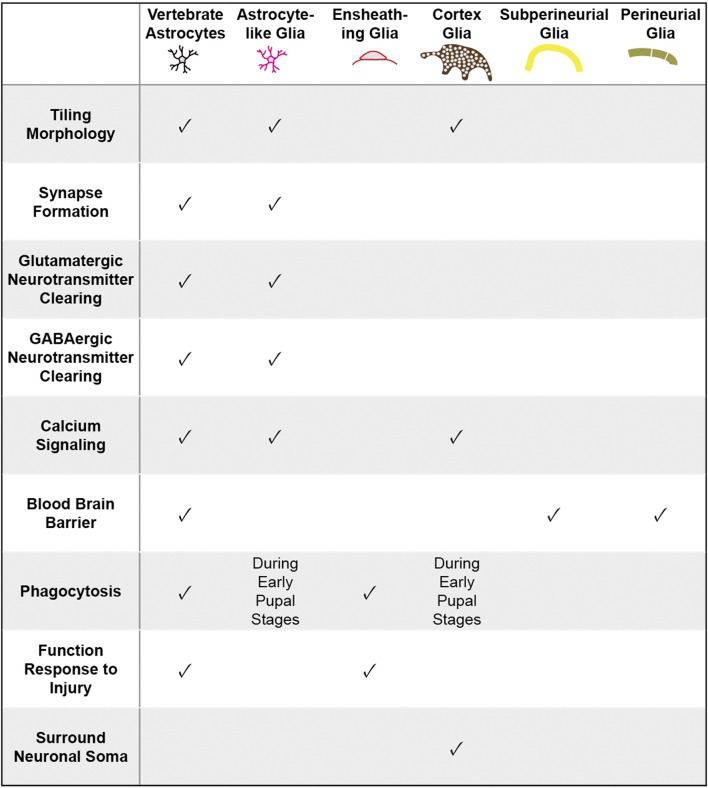

As in the rodent CNS, glial progenitors in *Drosophila* are also specified during embryonic development. Neuroblasts (NB) delaminate from the epithelium and generate neurons and glia of the larval CNS through neuron or glia-restricted neuroblasts or glioblasts, respectively, or bipotent neuroglioblasts, generating both neurons and glia (Hartenstein, 2011). A key determinant of glial cell fate is glial cells missing (*gcm*), a major effector for *Drosophila* glial specification. Loss of function of *gcm* perturbs differentiation of embryonically and postembryonically derived glia, while ectopic expression of *gcm* produces excess glia at the expense of neurons (Hosoya et al., 1995; Jones et al., 1995; Vincent et al., 1996). As in vertebrates, *Drosophila* glial cell fate is established through suppression of pro-neuronal genes and upregulation of pro-glial genes. In *Drosophila*, this is mediated through transcription factors *repo*, *pointed* and *tramtrack* that act downstream of *gcm* (Halter et al., 1995; Giesen et al., 1997; Badenhorst, 2001; Yuasa et al., 2003). Interestingly, the vertebrate homologs, *gcm1* and *gcm2*, are not required for glial specification in the mouse nervous system. Whereas these genes are easily detected in non-neural tissues of the developing mouse embryo, their expression in neural tissues is low (Kim et al., 1998). Nevertheless, expression of vertebrate *gcm1* or *gcm2* in *Drosophila* embryos is sufficient to promote glial fate and rescues *Drosophila gcm* mutants (Kim et al., 1998), highlighting the key role of this gene in *Drosophila* gliogenesis.

Notch is a conserved protein and similar to rodents, plays key roles in *Drosophila* glial specification and production. Notch signaling regulates the expression of *gcm* (Umesono et al., 2002). Mutants lacking Notch activity show a loss of glial cells (Ren et al., 2018) whereas ectopic activation of Notch activity produces an overabundance of glial cells (Udolph et al., 2001).

During metamorphosis, the *Drosophila* larval CNS is dramatically transformed through the combined actions of cell elimination, rewiring of connections and the production of new cells to generate the adult nervous system. Whereas the cellular and molecular mechanisms mediating neuron production are well characterized (Doe, 2017; Miyares and Lee, 2019), how glia of the adult CNS arise is not as well established. During late embryonic stages, NB that do not undergo apoptosis become quiescent but then resume proliferation in the larval stage, creating a second wave of neurogenesis responsible for 90% of neurons in the adult CNS (Truman and Bate, 1988; Homem and Knoblich, 2012; Lacin and Truman, 2016; Walsh and Doe, 2017; Harding and White, 2018). Similarly, glia progress through a second, post-embryonic stage of production and expansion to generate glia for the adult CNS (Hartenstein, 2011). This begins during the larval stage and is achieved through the proliferation of glial precursors, postembryonic NB, and differentiated glia in the larva (Pereanu et al., 2005; Omoto et al., 2015; Enriquez et al., 2018; Ren et al., 2018).

## *Drosophila* astrocyte-like glia (ALG) generation, migration, and morphogenesis

### ALG generation—Who am I?

While the key roles for *gcm* and Notch in glial cell fate specification are well established, the molecular determinants underlying specification of ALG and other classes of *Drosophila* glia remain poorly understood. Unlike vertebrate CNS development, the cellular lineage and final position of individual NBs is highly stereotyped and the NB lineages responsible for generating various classes of glial cells have been mapped (Beckervordersandforth et al., 2008; Altenhein et al., 2016). Neuropil glia arise from the longitudinal glioblast that generates longitudinal glia which differentiate into six ALG and three EG for each larval ventral nerve cord hemisegment (Jacobs et al., 1989; Stacey et al., 2007; Beckervordersandforth et al., 2008; Peco et al., 2016). Notch, in addition to its role in glial specification, drives embryonic ALG differentiation by regulating the expression of the homeodomain transcription factor prospero (*pros*) (Peco et al., 2016). Overexpression of a Notch repressor or knock down of *pros* leads to the loss of ALG markers including *Eaat1, Gat,* and *Ebony* and increase in the EG marker *Eaat2*, and ALG fail to infiltrate the neuropil.

While longitudinal glia have been identified as the embryonic precursors of larval neuropil glia, the precise source of ALG and EG found in the adult brain remains under debate. At the onset of metamorphosis, larval ALG begin to express *draper*, a gene necessary for phagocytosis, and adopt a spherical morphology similar to that of EG (Kato et al., 2020). It is currently hypothesized that larval ALG undergo apoptosis after performing phagocytosis of the degenerating larval nervous system (Tasdemir-Yilmaz and Freeman, 2014; Omoto et al., 2015; Pinto-Teixeira et al., 2016). This is supported by lineage tracing studies demonstrating that embryonically generated ALG soma are observed during early stages of pupal development, but are rare by 72 h after pupal formation (APF) and completely absent at 96 h APF (Omoto et al., 2015), suggesting these cells are eliminated before eclosion. TUNEL staining of GAT-positive cells demonstrates that ALG undergo programmed cell death (Omoto et al., 2015). Using an intersectional lineage tracing strategy, postembryonic Type II NB were identified as a source of adult ALG (Figure 2; Omoto et al., 2015; Enriquez et al., 2018; Ren et al., 2018). Type II NB produce intermediate neural progenitors (INPs) and rapidly expand the population of neurons and glia. Type II NBs can be multipotent, generating neurons, ALG, and EG (Ren et al., 2018). In addition to Type II NBs as a source of adult ALG, recent evidence demonstrates that some larval EG dedifferentiate to then generate adult ALG and EG during metamorphosis (Kato et al., 2020). However, to date, the exact contribution of embryonic and larval NBs to the full complement of adult ALG has not been definitively established. It is possible that different regions use differing strategies for generating the adult ALG population. In support of this, lineage tracing of Type II-derived ALG are found predominantly in the core of the brain whereas peripheral brain regions remain largely unlabeled (Ren et al., 2018). In addition, molecular signatures for lineage-specific identification of ALG precursors have not been well established. Because larval and adult flies exhibit distinct motor behaviors and underlying circuits, understanding how ALG in each of these distinct stages are generated and incorporate into neural circuits will be an important step towards understanding the development and function of neural circuits driving behavior.

Type II neuroblasts produce postembryonic INPs capable of generating neurons or glia (Urbach and Technau, 2003; Viktorin et al., 2011). Like vertebrate neural progenitor cells, multipotent Type II NBs generate glia following neuron production (Ren et al., 2018). Notch regulates gliogenesis from INPs which generate glial precursors that then undergo an expansion period during pupal stages before differentiating into adult ALG (Viktorin et al., 2011; Omoto et al., 2015). The mechanisms for INP expansion and neuropil glia differentiation are not well established, however, recent studies have again pinpointed Notch signaling as a major regulator of expansion (Ren et al., 2018).

### ALG migration—How did I get here?

How ALG localize to their positions surrounding the neuropil is also incompletely understood. In contrast to vertebrate astrocytes, the migration and position of neuropil glia precursors in *Drosophila* embryos is highly stereotyped. Glial precursors migrate to precise positions within the ventral nerve cord and brain preceding their differentiation into larval ALG and EG (Ito et al., 1995; Hartenstein et al., 1998; Beckervordersandforth et al., 2008; Omoto et al., 2015). Adult ALG also appear to have distinct migration patterns linked to their type II neuroblast origins, often incorporating with the neural cell types generated by the same neuroblast (Viktorin et al., 2011; Omoto et al., 2015; Ren et al., 2018). The mechanisms underlying migration patterns and the existence of location dependent molecular signatures remain relatively unexplored. The highly stereotyped manner by which ALG precursors migrate to their final positions suggest intrinsic molecular cues that instruct this process. The identification of such cues may provide insight into how vertebrate astrocytes find their way to their final positions in the CNS and how this process is coordinated with local environmental cues.

### ALG morphogenesis—How do I look?

A distinguishing characteristic of both *Drosophila* ALG and vertebrate astrocytes is their complex, bushy-like morphology comprised of fine processes that interact intimately with synapses. These processes tile the neuropil, establishing discrete territories of synapses within reach of an individual cell (Freeman, 2015). How these elaborate morphologies are established and maintained is only beginning to be understood. Similar to mammalian astrocytes, ALG morphogenesis coincides with synaptogenesis and the refinement of developing neural circuits. ALG processes are observed infiltrating the neuropil as early as late stage embryos (Stork et al., 2014). Morphogenesis of larval ALG cells is strongly regulated by the FGF receptor Heartless (Htl) (Stork et al., 2014). Loss of Htl does not disturb ALG specification, but results in failure of ALG invasion into the neuropil. Two key FGFs, Pyramus (Pyr) and Thisbe (Ths), activate Htl and promote morphogenesis and neuropil invasion. In conjunction with FGF signaling, *lapsyn*, a member of the extracellular leucine-rich repeat superfamily, is involved in ALG neuropil invasion and branch morphogenesis (Richier et al., 2017). Although ALG processes are observed into late larval stages and at puparium formation, they are absent from the neuropil by 48 h APF (Tasdemir-Yilmaz and Freeman, 2014). However, by 60 h APF, ALG processes are once again observed in the neuropil, and continue to elaborate their processes until 84 h APF, a time course coincident with synapse formation (Muthukumar et al., 2014). The signals that initiate invasion of ALG processes into the neuropil in the embryo or during pupal stages are not known. In addition, whether the seemingly distinct processes of neuropil invasion and branch morphogenesis are differentially regulated remain to be explored. Whereas the highly stereotyped manner by which *Drosophila* ALG are generated differs markedly from vertebrate astrocyte production, the remarkable parallels between *Drosophila* and vertebrates in astrocyte morphogenesis and its coordination with synaptogenesis offer an exciting opportunity to leverage discoveries from *Drosophila* for advancing fundamental principles of astrocyte development. Indeed, the identification of FGF receptor signaling as an important regulator of astrocyte morphogenesis in both *Drosophila* and vertebrates suggests that additional molecular cues may be conserved (Table 2).

**TABLE 2:** Key molecular and cellular mechanisms across various stages in vertebrate and *Drosophila* astrocyte-like glia development.

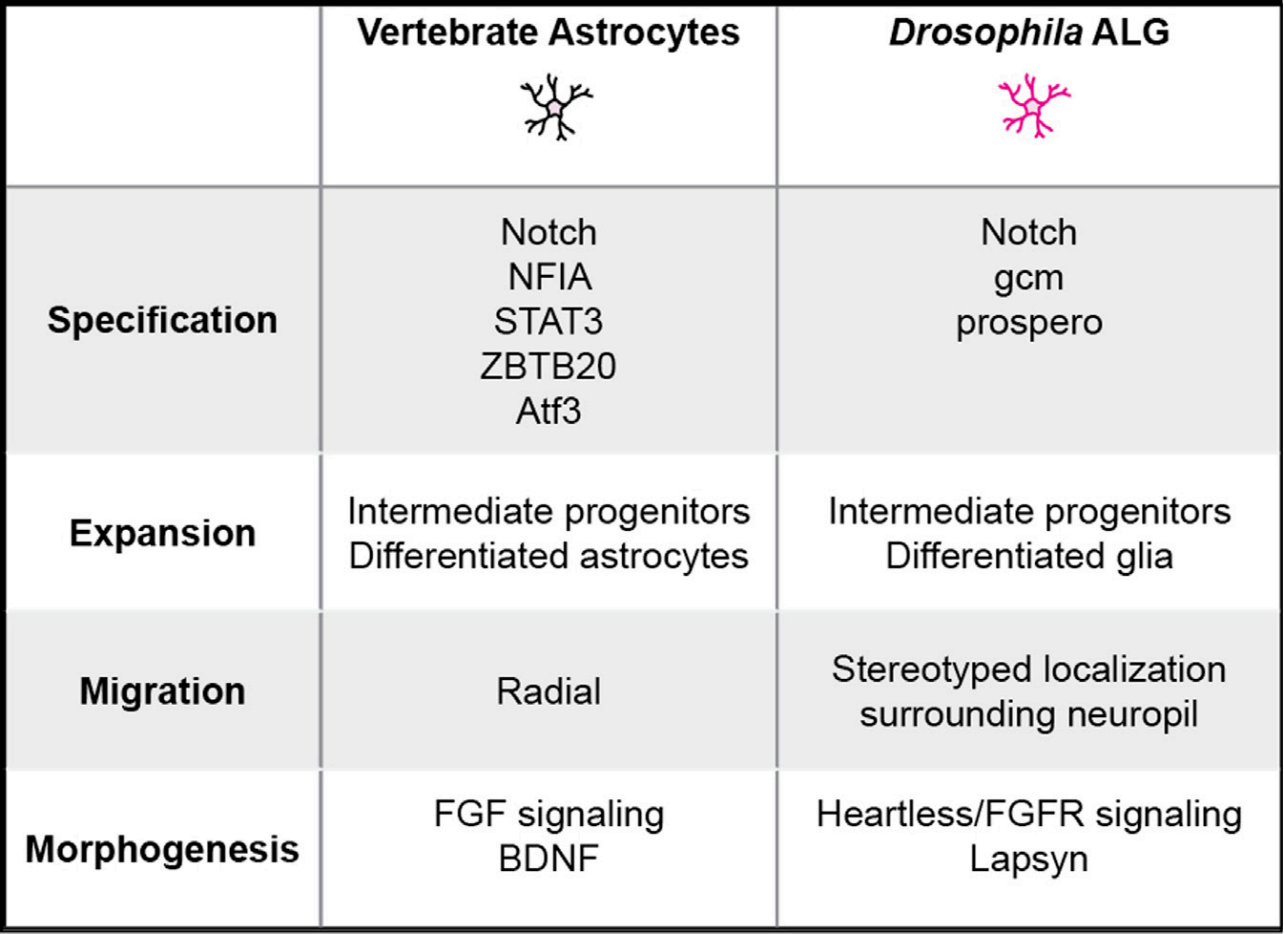

## How does astrocyte diversity arise?

Historically, astrocytes have been divided into two primary morphological classes, protoplasmic astrocytes found in gray matter and fibrous astrocytes in white matter. Yet the abundance of these cells in the CNS, together with the recognition of their diverse functional roles, has prompted great interest in their molecular and functional diversity. The emergence of RNA sequencing and bioinformatic analysis has enabled the discovery of transcriptionally distinct populations of astrocytes throughout the CNS. Such approaches have been applied most fervently in mouse tissues. However, emerging studies in *Drosophila* have also revealed previously unappreciated diversity of glia with defined transcriptional signatures. The nature of this heterogeneity and how it arises remain poorly understood.

In the mouse, a remarkable degree of molecular diversity has been demonstrated among astrocytes between regions, as well as within a region (Lanjakornsiripan et al., 2018; Hill et al., 2019; Batiuk et al., 2020; Bayraktar et al., 2020). In depth discussion of astrocyte heterogeneity in vertebrates is available from several excellent recent reviews and we refer the reader to these (Ben Haim and Rowitch, 2017; Khakh and Deneen, 2019; Pestana et al., 2020). An important question that arises from the discovery of these genetically defined populations is the role of intrinsic versus externally derived drivers of these molecular signatures.

Several studies demonstrate that neuronally derived cues regulate astrocyte gene expression. In the cerebellum, Sonic hedgehog (Shh) derived from Purkinje neurons regulates expression of genes in nearby Bergmann glia, such as GluA1, GluA4, GLAST and Kir4.1 (Farmer et al., 2016). Ectopic activation of Shh signaling in velate astrocytes, a morphologically and regionally distinct astrocyte population in the cerebellum that expresses low levels of these genes, increases expression of these and other genes associated with Bergmann glia. In the forebrain, mutants with aberrant patterning of cortical layers disrupts gene expression and distribution of morphologically defined astrocytes in a manner that follows the perturbed neuronal layers (Lanjakornsiripan et al., 2018; Bayraktar et al., 2020). These studies demonstrate remarkable plasticity in astrocyte phenotype and suggest that local, neuronally derived cues can drive molecular phenotypic diversity.

However, fate mapping studies provide evidence that developmental origin also plays a role in generating diversity. In the spinal cord, white matter astrocytes exhibit combinatorial expression of Reelin and Slit1 (Hochstim et al., 2008). These cells are derived from distinct progenitor cell domains in the ventricular zone defined by Pax6 and Nkx6.1. Similarly, protoplasmic astrocytes in the forebrain can be fate mapped from ventral, intermediate and dorsal progenitor domains expressing Nkx2.1, Dbx, and Emx1, respectively (Tsai et al., 2012). In both the spinal cord and forebrain, astrocytes derived from these progenitors occupy specific territories directly overlying these domains, in a manner suggesting that astrocytes undergo radial, but not tangential, migration. Notably, ablation of cells from one domain failed to recruit astrocytes from a different domain (Tsai et al., 2012), suggesting that lineage confers regional identities that are fixed. While these studies demonstrate regional specialization of astrocytes, evidence is emerging for diversity within a region.

In the spinal cord, a subpopulation of gray matter astrocytes express *Olig2* (Ohayon et al., 2019). These cells are derived from a subpopulation of *Olig2*-expressing precursor cells in the pMN domain that are regulated by expression of the heparin sulfate modification enzyme, *Sulf2*. *Olig2*-expressing astrocytes are intermixed among non*Olig2* astrocytes in the gray matter of the spinal cord, resulting in a heterogeneous mix of cells derived from a molecularly defined lineage. RNASeq identified specific molecular signatures of the *Olig2* and non*Olig2* astrocyte populations, including enrichment of *inka2* and *kcnip3* in *Olig2* astrocytes (Ohayon et al., 2021). In the mouse forebrain, recent studies demonstrated that activity of the Sonic hedgehog (Shh) signaling pathway occurs in a subpopulation of dlSVZ progenitors (Tong et al., 2015; Gingrich et al., 2022). These cells are identified by expression of the transcription factor, *Gli1*, a readout of Shh signaling. Using fate mapping approaches, Gingrich et al., demonstrated that these cells migrate out of the SVZ and proliferate locally in the cortex, undergoing rapid expansion early in the first postnatal week. Whether these cells correspond to “pioneer” cells with mature characteristics is not known. Astrocytes within the Gli1 lineage comprise nearly half the total cortical astrocyte population and are found throughout all layers, suggesting that the cortex harbors astrocytes from two molecularly defined astrocyte progenitor lineages. Consistent with this, single cell RNA sequencing (scRNA-seq) identified two molecularly distinct populations of astrocyte progenitor cells in the rodent forebrain (Liu et al., 2022). And using clonal analysis, homogenous clones of protoplasmic and fibrous astrocytes were observed in the cortex and corpus callosum, respectively, suggesting distinct lineages for morphologically defined astrocyte populations (Garcia-Marques and Lopez-Mascaraque, 2013). Whether and how these molecularly and morphologically defined lineages intersect remains to be discovered.

Taken together, these initial studies suggest that both lineage and local cues work in concert to define astrocyte characteristics. It is likely that molecular signaling programs initiate a transcriptional landscape that confers competence to interpret local cues in specific ways. What is the identity of these molecular signaling programs and how do they instruct cell identity? How plastic or fixed is astrocyte identity and how do these identities correspond to functional specialization? Early studies have begun to address these questions, but further efforts are needed to elucidate the nature of astrocyte heterogeneity and how it emerges.

There is also emerging evidence for greater heterogeneity among *Drosophila* glia than is currently appreciated. Although *Drosophila* glia have been classically organized into five classes defined by their distinct morphological and anatomical characteristics, recent transcriptomic atlases generated by scRNA-seq identified 19 transcriptionally distinct clusters of glial cells in the optic lobes of pupae (Kurmangaliyev et al., 2020). Remarkably, an independent study similarly identified 19 glial clusters from the adult optic lobe (Ozel et al., 2021). The convergence of 19 glial clusters from two independent studies across two life-cycle stages suggests that glia diversity may arise as a function of lineage. Indeed, *Drosophila* ALG demonstrate region specific morphological differences, particularly in the optic lobes of the adult brain (Edwards et al., 2012). Studies are needed to determine whether these morphological phenotypes correspond to specific transcriptional signatures. Type II neuroblasts reliably generate defined, molecularly and morphologically distinct neuronal cell types (Bayraktar and Doe, 2013; Ren et al., 2018), however, neuroblast specific signatures in ALG have yet to be uncovered. Alternatively, the clusters may reflect different physiological states of cells within a given class. ALG establish territories that are highly stereotyped across flies (Ren et al., 2018), but are still able to compensate for the loss of a lineage in both neuropil coverage and final cell counts (Stork et al., 2014; Richier et al., 2017), highlighting that environmental cues may also play a substantial role in within-class heterogeneity. Lineage tracing and functional studies are needed to distinguish between these possibilities.

## Future directions

It is remarkable to consider how far behind we are in our understanding of fundamental principles of astrocyte development, relative to what is known about development of myelinating cells and neurons. Across each stage of development lie many unanswered, yet basic, questions. What is the identity of intermediate astrocyte progenitor cells and how can they be distinguished from their more primitive parent cells? What are the cues that instruct the localization and distribution of astrocytes? How do intrinsic and extrinsic signals coordinate to drive the maturation and adoption of specific phenotypic and functional identities of astrocytes?

To date, rodent models have been predominant in the study of astrocytes. However, the emerging recognition of cells with key structural and functional properties of astrocytes in other model organisms, including zebrafish (Chen et al., 2020) and *Drosophila* (Stork et al., 2014), offers an opportunity to leverage the unique advantages of different models for complementary and parallel studies to advance our understanding of this historically understudied cell. *Drosophila*, in particular, due to their relatively inexpensive maintenance and short generation times, together with an abundance of existing highly sophisticated genetic tools including RNAi and intersectional tools for single cell manipulations, can be a workhorse for mechanistic discoveries.

While interrogating and manipulating astrocyte progenitor cells remains challenging due to insufficient tools and markers, the advanced molecular, genetic and imaging tools now available nevertheless afford opportunities for building on the existing foundations established by early neuroanatomical and molecular studies. In particular, the emergence of sequencing technologies opens the door to such discoveries and a growing number of transcriptional profiles of astrocytes from both *Drosophila* and mouse brains are now available for interrogation. Transcriptional profiling identified over 3,000 genes enriched in larval ALG (Huang et al., 2015). Notably, among these genes are those involved in cell morphogenesis and synapse development and function. Comparison with a comparable mouse study identified mouse homologs for nearly half of the larval-enriched genes, a majority of which are enriched in astrocytes (Huang et al., 2015). Together with the more recently published single cell transcriptional atlases of pupal development (Kurmangaliyev et al., 2020), and adult brains (Ozel et al., 2021), it is now possible to identify and interrogate the transcriptional signatures of glial cells across development. Identifying and mapping the *in vivo* expression of these genes may illuminate molecular and functional diversity that remains poorly understood. Single cell labeling using multi-color flip-out approaches and gene deletion approaches, for example, could facilitate analysis of morphological phenotypes and illuminate functional significance of these genes. Because many neural circuits and their behavioral outputs are well characterized in *Drosophila*, such targeted analyses provide an opportunity for dissecting how astrocytes modulate circuit function and behavior. There are also a growing number of transcriptomic atlases of the mouse brain, astrocyte-specific or including other cell types, across developmental stages and under various environmental conditions (Zhang et al., 2014; Hrvatin et al., 2018; Saunders et al., 2018, among many others). While many of these are restricted to cortical regions, there is nevertheless an unprecedented opportunity to identify novel molecular candidates that are conserved between species. Accompanied by lineage tracing and functional analysis, such studies will yield important novel insights into astrocyte development.
